# Familial partial lipodystrophy type 2 associated with a novel LMNA variant (c.604G>C; p.Glu202Gln): a Colombian family case series

**DOI:** 10.3389/fendo.2026.1788846

**Published:** 2026-04-10

**Authors:** Carolina Mendoza, Raquel Cano, Luis Burgos, Carlos Arturo Silvera

**Affiliations:** 1 Endocrinology Service, Clínica Virrey Solis, Barranquilla, Colombia; 2 Endocrinology Service, Clínica General del Norte, Barranquilla, Colombia; 3 Endocrinology Service, Clínica Portoazul, Barranquilla, Colombia; 4 Genetic Service, Clínica General del Norte, Barranquilla, Colombia

**Keywords:** case report, familial partial lipodystrophy type 2, LMNA gene, metabolic syndrome, variant of uncertain significance

## Abstract

**Introduction:**

Familial partial lipodystrophy type 2 (FPLD2) is a rare autosomal dominant laminopathy caused by LMNA gene variants. It is characterized by progressive gluteofemoral lipoatrophy and severe metabolic derangements, including insulin resistance and metabolic dysfunction–associated steatotic liver disease.

**Methods:**

Three Colombian women (two sisters and a daughter) underwent standardized clinical phenotyping, dual-energy X-ray absorptiometry (DXA), cardiometabolic laboratory testing, and next-generation sequencing–based testing for lipodystrophy-related genes with copy-number variant analysis.

**Results:**

All patients carried the heterozygous LMNA variant c.604G>C (p.Glu202Gln). Patients 1 and 2 exhibited classic Dunnigan phenotypes with diabetes and severe hypertriglyceridemia (up to 1,471 mg/dL), as well as imaging evidence of metabolic dysfunction–associated steatotic liver disease. Patient 3 (age 27) presented an evolving phenotype with central adiposity, clinically reported lower-limb fat loss, and insulin resistance (homeostatic model assessment for insulin resistance [HOMA-IR], 4), without hepatic steatosis on abdominal ultrasound. Management optimization in Patients 1 and 2 using contemporary combination therapy, including glucagon-like peptide-1 receptor agonist–based treatment and sodium-glucose cotransporter-2 inhibitors, was followed by sustained improvement in glycemia (HbA1c 11.1% to 7.6% in Patient 1) and triglycerides. In Patient 3, liraglutide was discontinued due to poor tolerability and limited weight response, and she was transitioned to semaglutide in February 2026.

**Conclusions:**

This case series expands the phenotypic spectrum of LMNA-associated FPLD2 and supports a disease association for the p.Glu202Gln variant despite its current classification as a variant of uncertain significance. It demonstrates that modern cardiometabolic therapies can be implemented in middle-income settings; however, the absence of functional data, population frequency metrics, and genetic testing in unaffected relatives limits the strength of variant interpretation.

## 1 Introduction

Familial partial lipodystrophy type 2 (FPLD2, Dunnigan type) is an autosomal dominant laminopathy characterized by progressive lipoatrophy of the limbs and gluteofemoral region with relative or absolute fat accumulation in the face, neck, and trunk, usually emerging around puberty (1, 2). Most cases are caused by heterozygous LMNA mutations, classically at codon 482 in exon 8, which disrupt adipocyte differentiation and storage capacity, promote ectopic fat deposition, and lead to insulin resistance, diabetes, severe hypertriglyceridemia, metabolic dysfunction–associated steatotic liver disease (MASLD), and increased cardiovascular risk (3, 4).

Estimated prevalence of partial and generalized lipodystrophy syndromes ranges around 1.7–2.8 per million, but underdiagnosis is substantial across clinical settings. Diagnostic pitfalls differ by sex and age: in younger women, the phenotype may be misattributed to polycystic ovary syndrome or common central obesity, whereas in other patients it may be dismissed as constitutional “muscularity” until severe metabolic complications emerge (5, 6). Newer next-generation sequencing (NGS) panels have expanded the catalog of LMNA variants, including multiple variants of uncertain significance (VUS), complicating genetic counseling (1, 7). Beyond adipose tissue disease, LMNA variants can cause a broader laminopathy spectrum involving cardiac conduction abnormalities, dilated cardiomyopathy, and skeletal muscle phenotypes (4, 8). This overlap is clinically relevant because patients diagnosed with FPLD2 may warrant proactive cardiovascular surveillance even when baseline testing is normal (9).

Therapeutic options have evolved beyond traditional insulin and fibrate therapy. Observational cohorts and case reports suggest that glucagon-like peptide-1 receptor agonists (GLP-1 RAs) and sodium-glucose cotransporter-2 inhibitors (SGLT2 inhibitors) can improve glycemia, triglycerides, and weight in patients with partial lipodystrophy (10, 11). Metreleptin is established for generalized lipodystrophy and may be considered in selected patients with partial forms who have severe metabolic complications despite optimized standard therapy; access varies widely across health systems (12, 13).

We describe three women from a Colombian family, two sisters and the eldest daughter of one sister, who harbor a novel LMNA c.604G>C (p.Glu202Gln) variant and display age-dependent phenotypic expression consistent with FPLD2. This report integrates clinical, body composition, cardiometabolic, and genetic data; follows CARE case-report standards; and illustrates pragmatic management in a middle-income setting where access to selected therapies and advanced testing may be administratively constrained (12, 14).

## 2 Patient information

### 2.1 Patient 1

A 51-year-old woman of mixed Afro-Colombian ancestry was referred in July 2024 for poorly controlled type 2 diabetes and a chronic plantar ulcer of the left foot. She was a homemaker living in an urban area with access to tertiary care. She recalled a “masculine” body habitus since preadolescence, with prominent musculature of the arms and legs despite not exercising. Menarche occurred at 12 years, followed by oligomenorrhea and marked hirsutism that persisted until perimenopause. Generalized myalgias and arthralgias had previously been labeled as fibromyalgia.

Type 2 diabetes was diagnosed at 44 years of age; complications included peripheral neuropathy and a chronic plantar ulcer. Additional history included mixed dyslipidemia and metabolic dysfunction–associated steatotic liver disease. At 41 years, she underwent total hysterectomy with unilateral oophorectomy for fibroids and ovarian cysts. She has three children: one daughter (Patient 3), who developed obesity, insulin resistance, and a body habitus that raised concern for familial partial lipodystrophy, and two younger sons, one deceased and one living, both reportedly asymptomatic and not genetically tested.

Family history suggested autosomal dominant inheritance. Her mother reportedly had similar fat redistribution, diabetes, hypertension, dyslipidemia, and breast cancer, and died of “cardiac” complications. One younger sister (Patient 2) had diabetes, mixed dyslipidemia, and the same body phenotype. The proband knew little about her maternal aunts and uncles but believed at least one maternal aunt had a similar appearance.

### 2.2 Patient 2

A 39-year-old woman, the younger sister of Patient 1, presented in April 2025 for evaluation of type 2 diabetes, hypertension, and severe hypertriglyceridemia. She also was a homemaker living in the same urban region. She recalled marked muscular hypertrophy of the extremities beginning at 9 years of age, preceding puberty.

Menarche occurred at 11 years, followed by two full-term pregnancies. Multiple ovarian cysts led to total hysterectomy with bilateral oophorectomy at age 33 years (two-stage procedure). Diabetes was diagnosed at 33 years; later she developed mixed dyslipidemia, neuropathic symptoms, and MASLD. She shared the same maternal family history as Patient 1.

### 2.3 Patient 3 (Patient 1’s daughter)

A 27-year-old woman, eldest daughter of Patient 1, was evaluated in May 2025 for suspected familial partial lipodystrophy. She lived in Barranquilla, was a homemaker, and had no children at the time of evaluation. Menarche occurred at 9 years. At approximately 24–25 years she experienced a spontaneous miscarriage at 12 weeks’ gestation. Around that time, she noted progressive increased muscle mass of the extremities, troublesome hirsutism, and strong axillary odor (bromhidrosis). She reported difficulty losing weight, with usual weight near 70 kg and a maximum of 84 kg despite dietary efforts.

History included left nephrectomy (indication not clearly documented), ovarian cysts, class 1 obesity, hirsutism, and acanthosis nigricans. There was no history of overt diabetes, pancreatitis, or cardiovascular events. She clearly recognized that her body habitus resembled her mother’s and aunt’s.

**Figure 1:**
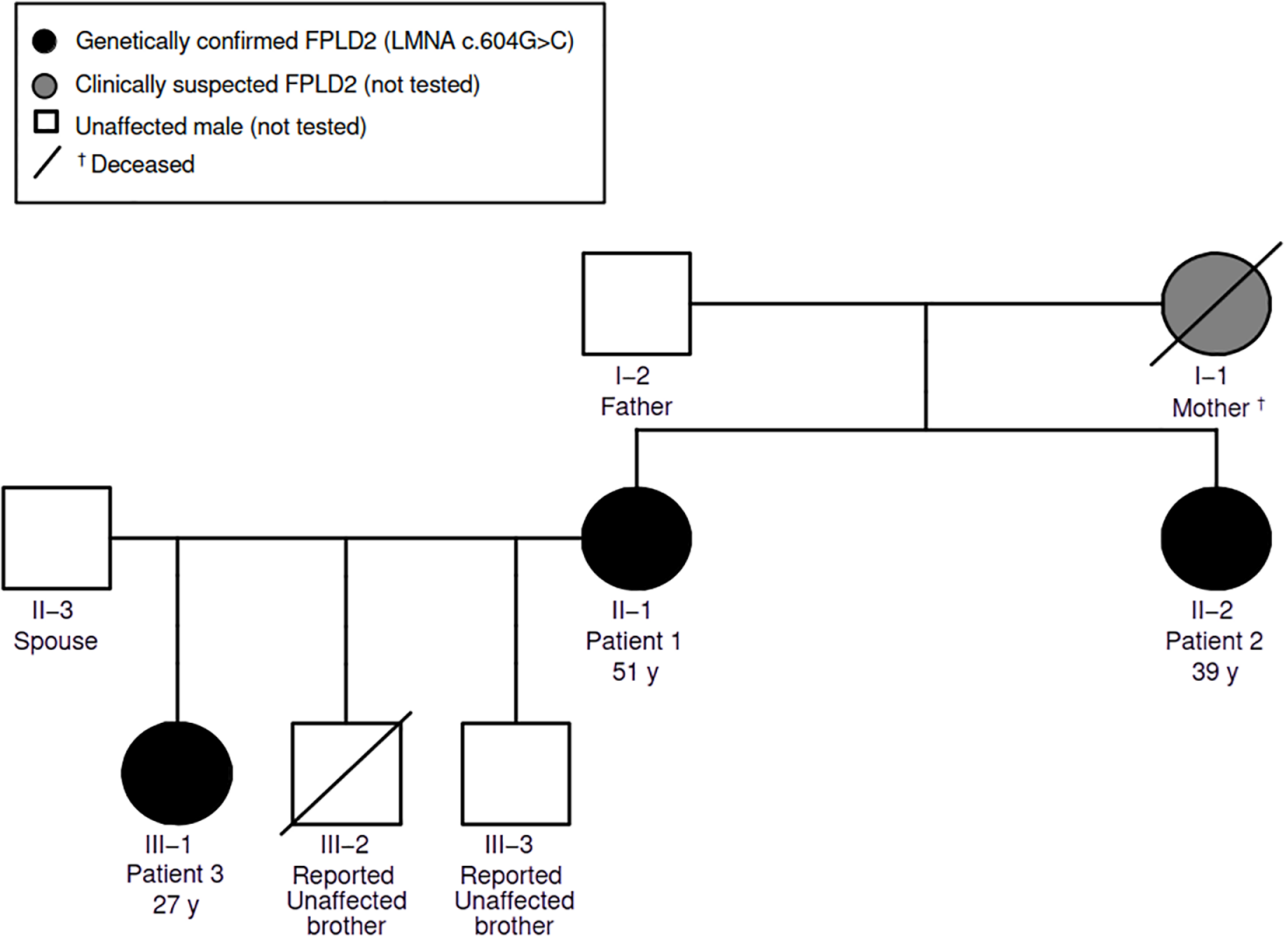
Pedigree of a Colombian family with LMNA-associated familial partial lipodystrophy type 2 (FPLD2). Filled symbols indicate genetically confirmed heterozygous carriers of LMNA c.604G>C (p.Glu202Gln) (Patients 1–3). The gray symbol indicates a clinically suspected affected relative who was not genetically tested. Open symbols indicate relatives who were not genetically tested; Patient 3’s two brothers were reported as unaffected (one deceased). A diagonal line denotes deceased status.

The family pedigree is consistent with autosomal dominant inheritance (**Figure 1**). The mother of Patients 1 and 2 was clinically suspected to be affected based on family report of similar fat redistribution and cardiometabolic disease but was not genetically tested. Patients 1–3 are affected and share the same LMNA c.604G>C (p.Glu202Gln) variant. Co-segregation is demonstrated among affected, tested relatives; unaffected relatives were not tested because genetic testing of asymptomatic family members is not routinely covered in our setting, which limits segregation strength and variant interpretation.

## 3 Clinical findings

### 3.1 Patient 1

**Figure 2:**
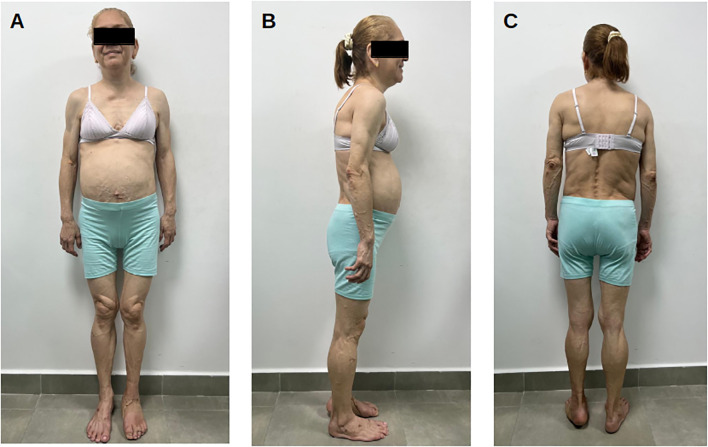
Clinical photographs of Patient 1 (51 years old). **(A)** Frontal view demonstrating marked lipoatrophy of upper and lower extremities with visible muscular hypertrophy, contrasting with preserved abdominal adiposity. Note visible superficial veins (phlebomegaly) in lower extremities. **(B)** Lateral view showing preserved truncal fat with complete absence of gluteal adiposity and dorsocervical fat pad (“buffalo hump”). **(C)** Posterior view demonstrating complete loss of gluteal and lower extremity subcutaneous fat with prominent muscular definition. Identity protected per ethical guidelines.

On examination, height was 160 cm, weight 55 kg, BMI 21.4 kg/m², waist circumference 80 cm, blood pressure 120/80 mmHg, and heart rate 72 beats/min. She had marked lipoatrophy of the arms, legs, and gluteofemoral region with preservation of palmar and plantar fat. Fat accumulation was evident in the face, neck (dorsocervical “buffalo hump”), scapular region, axillae, and mons pubis. Muscular hypertrophy and prominent superficial veins (phlebomegaly) were present in all limbs. Labia majora were hypertrophied. A chronic plantar ulcer was present without signs of acute infection. Acanthosis nigricans was not evident (**Figure 2**).

### 3.2 Patient 2

**Figure 3:**
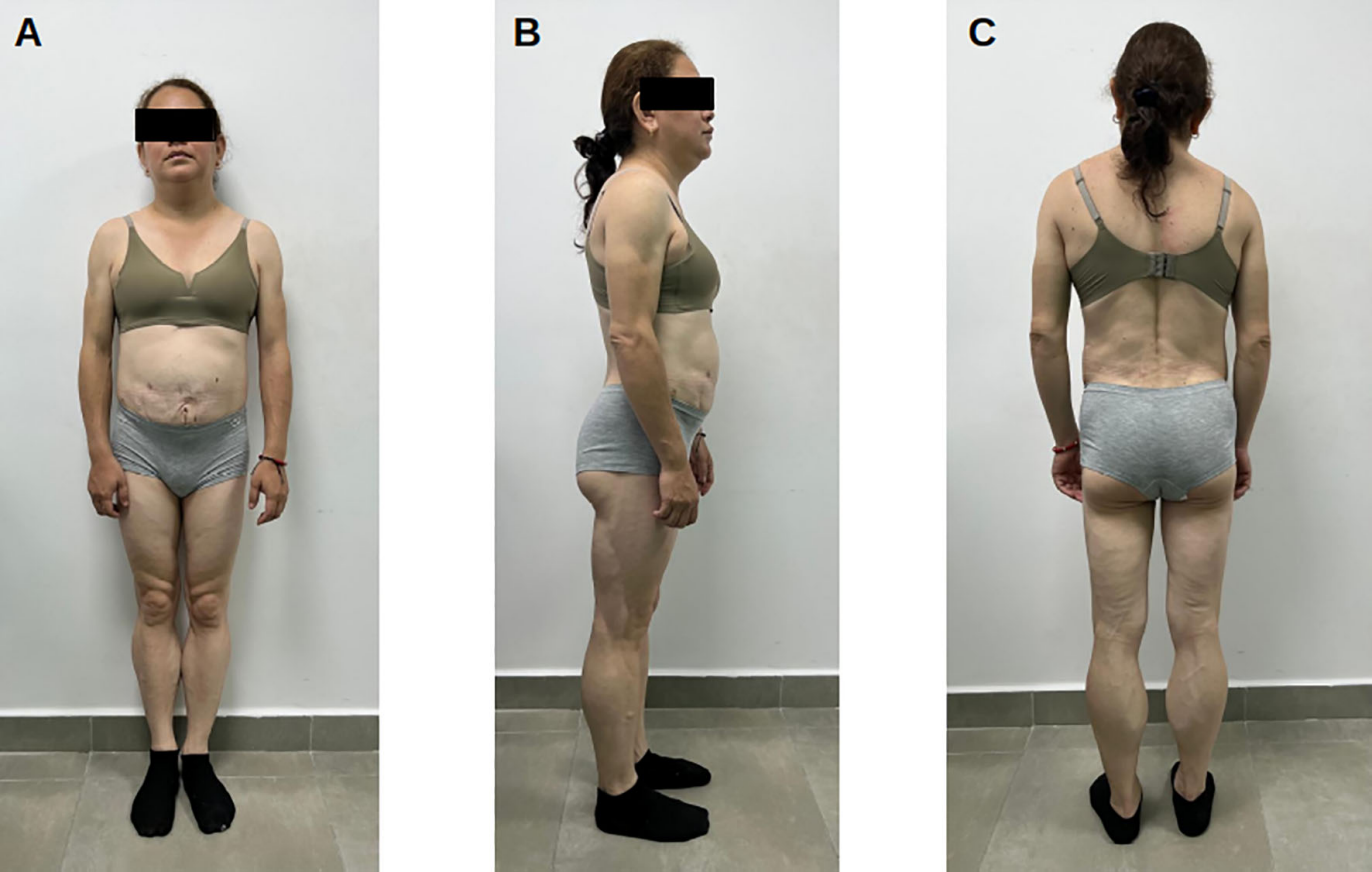
Clinical photographs of Patient 2 (39 years old). **(A)** Frontal view showing phenotype similar to Patient 1 with extremity lipoatrophy, muscular hypertrophy, and phlebomegaly. Preserved truncal adiposity evident. **(B)** Lateral view demonstrating characteristic contrast between truncal fat preservation and gluteofemoral lipoatrophy. **(C)** Posterior view showing absence of gluteal fat and prominent lower extremity muscularity with minimal subcutaneous tissue. Identity protected per ethical guidelines.

Height was 160 cm, weight 62 kg, BMI 24.2 kg/m², waist circumference 78 cm, and blood pressure 140/80 mmHg. Her phenotype closely resembled Patient 1: lipoatrophy of arms, legs, and gluteal region; preserved palmar and plantar fat; fat accumulation in neck, axillae, and scapular region; muscular hypertrophy; and phlebomegaly in all extremities. Labia majora were enlarged bilaterally. The degree of limb lipoatrophy appeared slightly less severe than in Patient 1 (**Figure 3**).

### 3.3 Patient 3

**Figure 4:**
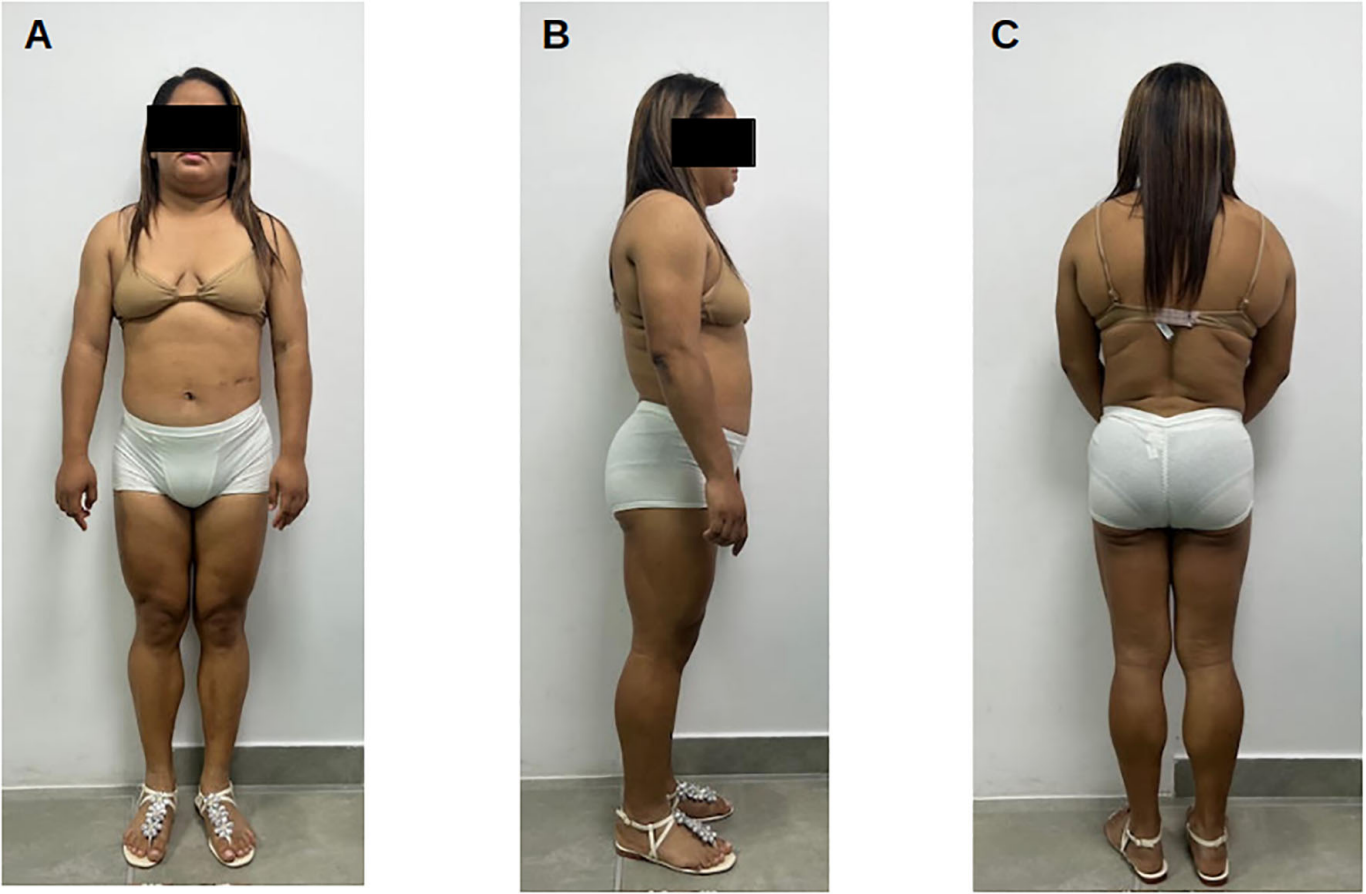
Clinical photographs of Patient 3 (27 years old). **(A)** Frontal view showing central adiposity with relative lower-limb muscularity and clinically suspected lower-limb subcutaneous fat loss. **(B)** Lateral view illustrating truncal adiposity in an early/attenuated partial lipodystrophy phenotype. **(C)** Posterior view showing reduced gluteofemoral subcutaneous fat relative to the trunk. Identity protected per ethical guidelines.

At evaluation, height was 165 cm and weight 84 kg (body mass index 32.8 kg/m², class I obesity). Physical examination documented increased relative muscle mass of the extremities, hirsutism, and acanthosis nigricans. The distribution pattern suggested an early or attenuated partial lipodystrophy phenotype: central adiposity with clinically reported lower-limb subcutaneous fat loss, without phlebomegaly at the time of assessment. Labia majora were enlarged bilaterally. No stigmata of Cushing syndrome were observed (**Figure 4**).

## 4 Timeline

**Table 1** summarizes the main life-course and clinical events for the three women.

**Table 1:** Comparative clinical timeline for the three affected family members.

Time period/age	Patient 1 – Mother (51 years)	Patient 2 – Aunt (39 years)	Patient 3 – Daughter (27 years)
Childhood	Pre-adolescent onset of “masculine” body habitus; muscular extremities without exercise	Age 9: marked muscular hypertrophy of extremities	History of early weight gain; detailed childhood body habitus not documented
Adolescence	Menarche 12 y; oligomenorrhea and hirsutism; progressive limb lipoatrophy	Menarche 11 y; progressive fat redistribution	Menarche 9 y; progressive hirsutism and weight gain in late adolescence
Young adulthood	G3P3; generalized myalgias and arthralgias diagnosed as fibromyalgia	G2P2; recurrent ovarian cysts requiring surgery	Miscarriage at 12 weeks (~24–25 y); onset of “excess” muscle mass, hirsutism, bromhidrosis
30s–40s	41 y: hysterectomy + unilateral oophorectomy for fibroids/cysts; 44 y: T2D diagnosed; chronic plantar ulcer and neuropathy develop (2018–2023)	33 y: T2D diagnosed; hysterectomy + bilateral oophorectomy (two stages) for ovarian cysts; hypertension and dyslipidemia develop	Class 1 obesity (BMI 32.8 kg/m²), insulin resistance, dyslipidemia; left nephrectomy and ovarian cysts documented

CAD-RADS, Coronary Artery Disease Reporting and Data System; CGM, continuous glucose monitoring; CTA, computed tomography angiography; DXA, dual-energy X-ray absorptiometry; HOMA-IR, Homeostatic Model Assessment for Insulin Resistance; LDL-C, low-density lipoprotein cholesterol; TG, triglycerides; TIR, time in range; T2D, type 2 diabetes.

## 5 Diagnostic assessment

### 5.1 Laboratory and imaging

#### 5.1.1 Laboratory and imaging – Patient 1

Baseline laboratory results (July 2024) showed marked metabolic derangement: HbA1c 11.1%, fasting glucose 138 mg/dL, triglycerides 429 mg/dL, total cholesterol 268 mg/dL, LDL-C 167 mg/dL, HDL-C 35 mg/dL. Serum creatinine was 0.77 mg/dL, and urine albumin–creatinine ratio (ACR) 17 mg/g. ALT and AST were 21 and 14 U/L, respectively. Thyroid function tests and inflammatory markers were unremarkable, and autoimmune screens (ANA, rheumatoid factor) were negative.

Hormonal studies confirmed postmenopause (FSH 62 mIU/mL, estradiol 5 pg/mL). Total testosterone (0.03 ng/mL), 17-hydroxyprogesterone, androstenedione, and DHEAS were all within or below reference ranges. IGF-1 was 185 ng/mL. Serum leptin was low-normal at 2.9 ng/mL (reference 3.7–11 ng/mL), consistent with partial leptin deficiency in the context of reduced subcutaneous fat.

Hepatobiliary ultrasound showed grade II hepatic steatosis. In our setting, ultrasound steatosis grade was reported by radiology based on hepatic echogenicity and sonographic attenuation relative to kidney cortex, consistent with routine clinical grading. Follow-up hepatobiliary ultrasound showed improvement from grade II to grade I steatosis after treatment optimization; elastography was not obtained.

DXA body composition revealed 22.6% total body fat (12.7 kg), lean mass 43.6 kg, visceral adipose tissue (VAT) 1,254 g, and an android-to-gynoid fat ratio of 2.0, confirming marked central fat predominance and near-complete lower-limb lipoatrophy consistent with an FPLD2 pattern (1, 14). ECG and transthoracic echocardiogram were normal, with left ventricular ejection fraction 60%. Carotid Doppler ultrasound showed fibrocalcific atheromatosis in the right carotid bulb with irregular heterogeneous plaques and fibrolipidic atheromatosis in the left carotid bulb with regular heterogeneous plaques, neither causing hemodynamically relevant stenosis. Coronary computed tomographic angiography was requested but not performed.

#### 5.1.2 Laboratory and imaging – Patient 2

At baseline (April 2025), HbA1c was 7.8%, creatinine 0.48 mg/dL, and ACR 163 mg/g, consistent with diabetic kidney disease. Triglycerides were 1,471 mg/dL, total cholesterol 268 mg/dL, HDL-C 39 mg/dL, and LDL-C 62 mg/dL. ALT was 30 U/L, AST 24 U/L, GGT 24 U/L, and alkaline phosphatase 69 U/L. Thyroid function was normal.

She had biochemical diagnosis of menopause (FSH 39 mIU/mL, estradiol <24 pg/mL). Leptin was 5.4 ng/mL, within the reference range but inappropriately low for her BMI, reflecting limited functional adipose tissue (3).

Ultrasound showed grade III hepatic steatosis with hepatomegaly. FibroScan yielded a median stiffness of 4.7 kPa, supporting a low probability of advanced fibrosis. Follow-up liver ultrasound after treatment intensification described mild steatosis.

DXA demonstrated 25.3% total body fat (14.5 kg), lean mass 42.8 kg, VAT 806 g, and android-to-gynoid ratio 1.64, again indicating disproportionate central fat with marked limb lipoatrophy. ECG and echocardiogram were normal (LVEF 65%), and coronary CTA showed CAD-RADS 0.

#### 5.1.3 Laboratory and imaging – Patient 3

Between January 2024 and April 2025, HbA1c rose from 5.9% to 6.3%, while fasting glucose remained <90 mg/dL and postprandial glucose approximately 126 mg/dL. Triglycerides ranged from 138–164 mg/dL, total cholesterol was 203 mg/dL, LDL-C 172 mg/dL, and HDL-C 29 mg/dL. Fasting insulin was 48 μU/mL on one occasion; later samples showed insulin 21.74 μU/mL with HOMA-IR of 4, confirming insulin resistance. Liver enzymes were within normal ranges. Urine microalbumin was 18.3 μg/mL (no overt albuminuria).

Hormonal testing demonstrated androgen levels consistent with hyperandrogenism: total testosterone 0.22 ng/mL with low SHBG (22.14 nmol/L, reference 32.4–128), free testosterone 0.09 pg/mL, and DHEAS 1.50 μg/mL. 17-hydroxyprogesterone and ACTH were normal.

DXA (March 2025) showed 28.3% total body fat (22.36 kg), lean mass 56.5 kg, VAT 1,372 g, android-to-gynoid ratio 1.24, and relative skeletal muscle index 9.63 kg/m². These data demonstrate central fat accumulation coupled with relative limb lipoatrophy in the context of obesity, a pattern compatible with early FPLD2 (2, 4). Abdominal ultrasound documented an increased size right kidney with nephrolithiasis, absent left kidney (post-nephrectomy), and left ovarian cysts. Abdominal ultrasound did not demonstrate hepatic steatosis or other chronic liver disease. Thoracic vascular CT angiography identified a small Stanford type B dissection flap in the proximal descending aorta and an anatomic type II aortic arch variant. A pharmacologic stress myocardial perfusion test was negative for inducible ischemia.

### 5.2 Comparative baseline characteristics

Key baseline anthropometric, biochemical, and body composition data are summarized in **Table 2**.

**Table 2:** Baseline clinical and biochemical characteristics.

Parameter	Patient 1 – Mother (51 years)	Patient 2 – Aunt (39 years)	Patient 3 – Daughter (27 years)	Reference ranges
Height (cm)	160	160	165	—
Weight (kg)	55	62	84	—
BMI (kg/m²)	21.4	24.2	32.8	18.5–24.9
Waist (cm)	80	78	Not available	<88 (women)
Blood pressure (mmHg)	120/80	140/80	Not available	<130/80
HbA1c (%)	11.1	7.8	6.3	<5.7; <7.0 (goal in diabetes)
Fasting glucose (mg/dL)	138	96	88	70–100
Triglycerides (mg/dL)	429	1,471	138	<150
LDL-C (mg/dL)	167	62	172	<100 (diabetes)
HDL-C (mg/dL)	35	39	29	>50 (women)
Creatinine (mg/dL)	0.77	0.48	Not available	0.6–1.2
Urine ACR	17 mg/g	163 mg/g	Not available	<30 mg/g
Urine microalbumin (μg/mL)	Not available	Not available	18.3	—
MASLD on ultrasound	Grade II	Grade III	No steatosis	—
DXA total fat (%)	22.6	25.3	28.3	20–35
Android/gynoid ratio	2.0	1.64	1.24	<0.8
VAT (g)	1,254	806	1,372	<500
Leptin (ng/mL)	2.9	5.4	Not available	3.7–11.0
LMNA genotype	c.604G>C (p.Glu202Gln), het.	c.604G>C (p.Glu202Gln), het.	c.604G>C (p.Glu202Gln), het.	—

ACR, albumin-to-creatinine ratio; BMI, body mass index; DXA, dual-energy X-ray absorptiometry; HDL-C, high-density lipoprotein cholesterol; LDL-C, low-density lipoprotein cholesterol; MASLD, metabolic dysfunction–associated steatotic liver disease; US, ultrasound; VAT, visceral adipose tissue. Note: The available DXA reports did not include the regional trunk and leg fat percentages required to compute a standard trunk-to-leg fat mass ratio.

### 5.3 Genetic testing

Genetic testing was performed at Genecell (Colombia) using next-generation sequencing with copy-number variant (CNV) analysis for lipodystrophy-related genes. All three women carried the same heterozygous LMNA variant c.604G>C (NM_170707.4), predicted to result in a missense substitution p.(Glu202Gln) in exon 3/12, with variant allele fractions consistent with heterozygosity (approximately 0.54–0.60 across reports). No CNVs were detected in the analyzed genes. The laboratory reports noted that this variant was not previously reported in ClinVar, HGMD, or LOVD, and described population frequency as not established in gnomAD at the time of reporting. In silico prediction tools reported by the laboratory (including BayesDel addAF/noAF, REVEL, and MutPred) supported a deleterious effect, contributing to ACMG PP3 in the clinical interpretation. Using ACMG criteria cited in the reports (PM1, PM2, and PP3), the variant was classified as a VUS.

Accordingly, our data support a clinically meaningful association between the p.(Glu202Gln) variant and the observed phenotype in this family, while recognizing that formal reclassification beyond VUS requires additional evidence not available in this case series.

### 5.4 Diagnostic reasoning and differential diagnosis

The diagnosis of FPLD2 was supported by a highly coherent set of findings: a stereotyped caudocranial pattern of fat redistribution with limb and gluteofemoral lipoatrophy; DXA evidence of markedly increased android-to-gynoid fat ratios with elevated visceral adipose tissue; diabetes or prediabetes, dyslipidemia, and MASLD that were disproportionate to BMI; an autosomal dominant family history involving three women in successive generations; and a heterozygous LMNA missense variant that segregated with the phenotype. Taken together, these elements fulfilled current guideline criteria for FPLD2 (2, 12, 13).

Alternative diagnoses were considered, including other familial partial lipodystrophy subtypes and phenocopies. Genetic differentials include PPARG-related familial partial lipodystrophy type 3 and variants in other lipodystrophy-associated genes such as PLIN1, AKT2, CIDEC, PCYT1A, and PIK3R1, among others included in the tested panel. Acquired partial lipodystrophy (Barraquer–Simons) was unlikely given the absence of a cephalocaudal fat-loss pattern, complement abnormalities, or a compatible clinical trajectory, and there was no history of human immunodeficiency virus exposure or antiretroviral therapy. Endocrine mimics such as Cushing syndrome and acromegaly were not supported by clinical features or hormone testing. Polycystic ovary syndrome could explain aspects of hyperandrogenism in Patient 3, but it does not account for the familial clustering of lipoatrophy and the shared LMNA variant across affected relatives.

The clinical picture matched the spectrum of LMNA-associated FPLD described in multiple cohorts, including the variability in adipose distribution and metabolic severity across different LMNA mutations (1, 4, 14).

## 6 Therapeutic intervention

Management followed international lipodystrophy guidelines and standard diabetes/cardiometabolic care, tailored to each case (12, 13).

### 6.1 Patient 1

At referral she was using insulin glargine 20 U/day, insulin glulisine 6 U with meals, dapagliflozin 10 mg/day, sitagliptin/metformin 50/1,000 mg twice daily, and atorvastatin 10 mg/day with irregular adherence.

Therapy was reorganized in July 2024: transition to fixed-ratio insulin degludec/liraglutide (IDegLira) 18 U once daily (18 U degludec + 0.6 mg liraglutide), plus empagliflozin/metformin 12.5/1000 mg twice daily. Lipid management was switched to rosuvastatin/fenofibrate 20/135 mg daily. Neuropathy was treated with alpha-lipoic acid 1,200 mg/day and pregabalin 75 mg/day. Real-time continuous glucose monitoring (CGM) was introduced for titration and education. Metreleptin is available in Colombia but access is restricted by administrative and reimbursement barriers; given her only modest leptin reduction and the complexity of obtaining treatment authorization, it was not prioritized (12, 13).

### 6.2 Patient 2

At baseline she received sitagliptin/metformin 50/1000 mg once daily, enalapril 20 mg/day, and rosuvastatin 10 mg/day.

In April 2025, her regimen was intensified: dapagliflozin/metformin 10/1000 mg daily, rosuvastatin/fenofibric acid 20/135 mg daily to aggressively reduce triglycerides (pancreatitis risk at 1,471 mg/dL), and switch from enalapril to telmisartan 40 mg/day for renal protection. Neuropathy was managed with alpha-lipoic acid 1,200 mg/day and pregabalin 75 mg/day.

### 6.3 Patient 3

Liraglutide was initiated with dose escalation to target weight reduction and insulin resistance, with the clinical goal of reducing progression risk in a patient with prediabetes-range glycemia (10, 11). Spironolactone was escalated from 100 mg to 150 mg orally once daily for hirsutism and bromhidrosis, with reported symptom improvement. Orlistat was discontinued due to insufficient weight response.

### 6.4 Lifestyle and preventive measures (all patients)

All three women received structured counseling on moderate caloric restriction, low–glycemic index carbohydrates, increased fiber, limited saturated fat, and inclusion of monounsaturated and omega-3 fats. They were advised to perform ≥150 minutes/week of moderate aerobic activity plus resistance exercise twice weekly, adjusted for neuropathy and ulcer risk in Patient 1. All were nonsmokers.

## 7 Follow-up and outcomes

### 7.1 Patient 1

Glycemic and lipid evolution over 12 months is shown in **Table 3**.

**Table 3:** Evolution of key metabolic parameters in Patients 1 and 2.

Parameter	Patient 1 – Baseline (Jul 2024)	Patient 1–3 mo (Oct 2024)	Patient 1–6 mo (Dec 2024)	Patient 1–12 mo (Jun 2025)	1Patient 2 – Baseline (Apr 2025)	Patient 2–3 mo (Jul 2025)
HbA1c (%)	11.1	9.6	7.7	7.6	7.8	6.9
Triglycerides (mg/dL)	429	162	Not reported	150	1471	252
LDL-C (mg/dL)	167	61	Not reported	62	62	39
Total cholesterol (mg/dL)	268	113	Not reported	Not reported	268	Not reported
Creatinine (mg/dL)	0.77	0.62	Not reported	Not reported	0.48	Not reported
Treatment regimen at timepoint (key changes)	Insulin glargine + insulin glulisine + dapagliflozin + sitagliptin/metformin + atorvastatin (irregular adherence to rapid-acting insulin)	Switched to insulin degludec/liraglutide (IDegLira) + empagliflozin/metformin + rosuvastatin/fenofibrate; CGM initiated	Continued IDegLira + empagliflozin/metformin + rosuvastatin/fenofibrate; CGM used for titration	Continued IDegLira + empagliflozin/metformin + rosuvastatin/fenofibrate	Sitagliptin/metformin + enalapril + rosuvastatin	Switched to dapagliflozin/metformin + telmisartan + rosuvastatin/fenofibric acid

CGM, continuous glucose monitoring; LDL-C, low-density lipoprotein cholesterol. Note: Reported treatment regimens reflect real-world access and tolerability; interruptions in medication supply are noted where relevant (10, 11).

Three months after regimen optimization (October 2024), HbA1c decreased to 9.6%, LDL-C to 61 mg/dL, total cholesterol to 113 mg/dL, and triglycerides to 162 mg/dL. At six months (December 2024), HbA1c further improved to 7.7%.

CGM in January 2025 showed sensor use 79%, glucose management indicator (GMI) 6.9%, time in range (70–180 mg/dL) 81%, coefficient of variation 11%, time below range 0%, and time above range >180 mg/dL 24%—metrics consistent with excellent glycemic control and very low hypoglycemia risk. At 12 months (June 2025), HbA1c was 7.6%, LDL-C 62 mg/dL, and triglycerides 150 mg/dL, with stable weight (~55 kg).

Medication adherence was assessed as adequate based on prescription refill records and achievement of glycemic targets. No gastrointestinal adverse effects or episodes of severe hypoglycemia were documented in clinical records. Despite repeated referrals, she did not complete coronary imaging, an important unaddressed risk given the known association of LMNA mutations with cardiac disease (9, 12).

### 7.2 Patient 2

At three months (July 2025), HbA1c improved from 7.8% to 6.9%. LDL-C decreased from 62 to 39 mg/dL, and triglycerides from 1,471 to 252 mg/dL, representing approximately an 83% reduction. Weight remained stable at 62 kg. Treatment adherence was considered adequate based on prescription records and achievement of glycemic and lipid targets.

No adverse events were documented in clinical records. Echocardiography remained normal (LVEF 65%), and coronary CTA showed CAD-RADS 0, reassuring for the absence of early coronary plaque, although lifetime risk remains elevated given her underlying lipodystrophy and diabetes (9, 12).

### 7.3 Patient 3

Serial metabolic testing before initiation of GLP-1 RA therapy showed progressive dysglycemia within the prediabetes range (HbA1c 5.9% to 6.3%) and insulin resistance (fasting insulin 21.74 μU/mL with homeostatic model assessment for insulin resistance 4), with dyslipidemia characterized by low high-density lipoprotein cholesterol and elevated low-density lipoprotein cholesterol. Pharmacotherapy evolved in real-world fashion: metformin was previously discontinued due to gastrointestinal intolerance; lifestyle measures were implemented; spironolactone was initiated for hyperandrogenic symptoms; orlistat was stopped due to limited weight response; and liraglutide was prescribed for weight and insulin-resistance targets. At follow-up in February 2026, the patient reported minimal weight response and gastrointestinal adverse effects that limited tolerability on liraglutide, prompting a switch to semaglutide 2.4 mg weekly. No post–liraglutide cardiometabolic laboratory reassessment was available before the medication switch; therefore, clinical tolerability and self-reported weight response were the primary documented outcomes during that interval. Leptin testing was ordered but had not been completed at the time of manuscript revision.

## 8 Discussion

This family case series illustrates key aspects of LMNA-associated FPLD2, including age-dependent phenotypic expression and pragmatic use of contemporary cardiometabolic therapies in a middle-income setting.

Most FPLD2 cases carry LMNA variants in exons 8–11, especially p.Arg482 variants, but the spectrum associated with non-hotspot variants continues to expand (1, 4). The LMNA c.604G>C (p.Glu202Gln) variant identified here lies in exon 3 within the N-terminal head domain, a region relevant to lamin A/C assembly. Co-segregation among the affected, tested relatives and a compatible phenotype support a disease association; however, population frequency metrics, standardized computational prediction outputs, and functional assays were not available in the clinical report. For these reasons, the variant remains best described as a VUS with supportive clinical evidence rather than definitively reclassified on the basis of this case series alone.

(1, 4) (7, 8) (1, 7)The three women demonstrate how FPLD2 unfolds across the life course. The 27-year-old daughter has obesity, insulin resistance, and dyslipidemia but not yet overt diabetes or severe hypertriglyceridemia. Her 39-year-old aunt presents with diabetes, microalbuminuria, and life-threatening triglyceride levels (1,471 mg/dL), while her 51-year-old mother has longstanding poorly controlled diabetes, neuropathy, peripheral atherosclerosis, and chronic ulceration. This gradient reflects progressive ectopic lipid deposition and insulin resistance over time (3, 5).

DXA data quantify this process: all three have elevated android-to-gynoid ratios and increased VAT, but the youngest is the only one with obesity, illustrating that FPLD2 can coexist with generalized adiposity (2, 6). Recognition of this “hybrid” phenotype is essential in younger women with apparent central obesity and PCOS-like features.

Diagnostic guidelines emphasize a thorough physical examination focusing on fat distribution, early-onset metabolic syndrome, and family history when FPLD is suspected (12, 13). In large cohorts, LMNA-associated FPLD2 is characterized by marked extremity muscularity, prominent superficial veins, gluteofemoral lipoatrophy, and disproportionate metabolic disease (4, 14). Patients 1 and 2 closely matched this classic pattern, including phlebomegaly, whereas Patient 3 demonstrated a milder, age-dependent presentation with clinically reported lower-limb fat loss but without phlebomegaly at the time of assessment, consistent with variable expressivity (4, 14). In Latin America, limited access to specialized lipodystrophy centers, DXA, and NGS panels contributes to under-recognition (5). This family was initially managed for diabetes, dyslipidemia, obesity, and PCOS-like manifestations without a unifying diagnosis. Recognition of the index case’s phenotype led to DXA and genetic testing, followed by cascade screening identifying the younger relative early—the recommended approach for monogenic lipodystrophy (2, 13).

Management focuses on metabolic complications using intensive regimens (12, 13). GLP-1 RAs and SGLT2 inhibitors proved valuable in our family. A French cohort of 76 FPLD patients showed durable improvements in BMI, HbA1c, and triglycerides with GLP-1 RAs (10), while a pediatric FPLD2 case achieved glycemic control with liraglutide within two months (11). Consistent with these reports, Patient 1 experienced HbA1c reduction from 11.1% to 7.6% and substantial lipid improvements under IDegLira plus sodium-glucose cotransporter-2 inhibition. In Patient 3, GLP-1 RA therapy was initiated to target weight and insulin resistance, but tolerability limitations ultimately prompted transition to semaglutide.

SGLT2 inhibitors contributed to improved glycemic control, weight reduction, and renal protection (microalbuminuria in Patient 2). Fibrate–statin combinations were central to managing extreme hypertriglyceridemia, as recommended for severe dyslipidemia in lipodystrophy (12, 13).

Metreleptin is an established therapy for generalized lipodystrophy and may be considered in selected patients with partial lipodystrophy who have severe metabolic complications despite optimized standard therapy. Evidence does not support using baseline leptin concentration alone to predict clinical response. In our setting, access is often constrained by administrative and reimbursement barriers; therefore, management prioritized intensive cardiometabolic therapy using agents with clearer local availability and documented benefit in insulin resistance and hypertriglyceridemia.

(12, 13) (6, 10)LMNA mutations can cause cardiac laminopathy with conduction defects and cardiomyopathy (9). In FPLD2, premature atherosclerotic disease is common due to severe, chronic metabolic derangement (1, 14). The reported cardiac death of the maternal grandmother and peripheral vascular disease in Patient 1 underscore this risk. While Patient 2’s cardiac imaging is reassuring, long-term follow-up remains necessary. The incomplete cardiac evaluation in Patient 1 highlights a practical clinical lesson: LMNA-associated FPLD2 warrants proactive cardiovascular surveillance even when baseline testing is normal (9, 12).

Strengths include detailed phenotyping of two adult sisters with imaging and longitudinal cardiometabolic data, documentation of the same LMNA missense variant in three affected relatives, and description of contemporary treatment strategies in a middle-income setting. Limitations include absence of functional validation for the LMNA variant, unavailable population frequency and standardized in silico prediction outputs in the clinical report, incomplete longitudinal outcomes for Patient 3 after transition to semaglutide, and incomplete cardiovascular evaluation in Patient 1. Unaffected relatives were not sequenced, limiting the strength of segregation evidence, and historical photographs of deceased relatives were not available for publication due to family preference; therefore, disease status in earlier generations relies on family report. These limitations reflect routine care in a middle-income setting where repeat specialized testing is not consistently available.

In women with “muscular” limbs, gluteofemoral lipoatrophy, central fat accumulation, severe dyslipidemia, and insulin resistance disproportionate to BMI, especially with similar family history, FPLD2 should be considered. Careful phenotyping, targeted LMNA testing, and cascade screening can identify affected relatives early. LMNA variants outside codon 482, such as c.604G>C (p.Glu202Gln), can produce classic FPLD2 phenotypes. Modern combinations of GLP-1 RAs, SGLT2 inhibitors, and fibrate–statin therapy can substantially improve metabolic control, including in settings where metreleptin is difficult to access or not indicated.

## Data Availability

The raw data supporting the conclusions of this article will be made available by the authors, without undue reservation.
